# Autophagy of the Nucleus in Health and Disease

**DOI:** 10.3389/fcell.2021.814955

**Published:** 2022-01-03

**Authors:** Georgios Konstantinidis, Nektarios Tavernarakis

**Affiliations:** ^1^ Institute of Molecular Biology and Biotechnology, Foundation for Research and Technology - Hellas, Heraklion, Greece; ^2^ Department of Basic Sciences, School of Medicine, University of Crete, Heraklion, Greece

**Keywords:** ageing, autophagy, cancer, neurodegeneration, nucleophagy

## Abstract

Nucleophagy is an organelle-selective subtype of autophagy that targets nuclear material for degradation. The macroautophagic delivery of micronuclei to the vacuole, together with the nucleus-vacuole junction-dependent microautophagic degradation of nuclear material, were first observed in yeast. Nuclear pore complexes and ribosomal DNA are typically excluded during conventional macronucleophagy and micronucleophagy, indicating that degradation of nuclear cargo is tightly regulated. In mammals, similarly to other autophagy subtypes, nucleophagy is crucial for cellular differentiation and development, in addition to enabling cells to respond to various nuclear insults and cell cycle perturbations. A common denominator of all nucleophagic processes characterized in diverse organisms is the dependence on the core autophagic machinery. Here, we survey recent studies investigating the autophagic processing of nuclear components. We discuss nucleophagic events in the context of pathology, such as neurodegeneration, cancer, DNA damage, and ageing.

## Introduction

Autophagy is an evolutionarily conserved catabolic process enabling cells to maintain homeostasis as well as respond to stress conditions. Misfolded or aggregated proteins, damaged organelles and invading pathogens can be recognized by respective types of selective autophagy. Autophagic cargos are delivered to the vacuole/lysosomes in order to be degraded (Kroemer et al., 2010; Yang and Klionsky, 2010; Mizushima and Komatsu, 2011). Defects in autophagy have been linked to a wide range of pathological conditions, including cancer, neurodegeneration and inflammatory, metabolic and infectious diseases (Dikic and Elazar, 2018; Levine and Kroemer, 2019; Kawabata and Yoshimori, 2020). Deoxyribonucleic acid (DNA) damage or nuclear envelope dysfunction are associated with aberrant nuclear activity, dynamics and cell signaling (Mijaljica et al., 2010; Hauer and Gasser, 2017; Karoutas and Akhtar, 2021). Therefore, clearance of damaged nuclear components is crucial for the maintenance of the nuclear integrity. Nucleophagy is the degradation of nuclear material including nuclear membrane, nuclear lamina, nucleoplasm, nucleolus and DNA by the autophagic machinery. From yeast to humans, nucleophagic events are monitored in a species- and a context-specific manner.

### Nucleophagy in Yeast

**FIGURE 1:**
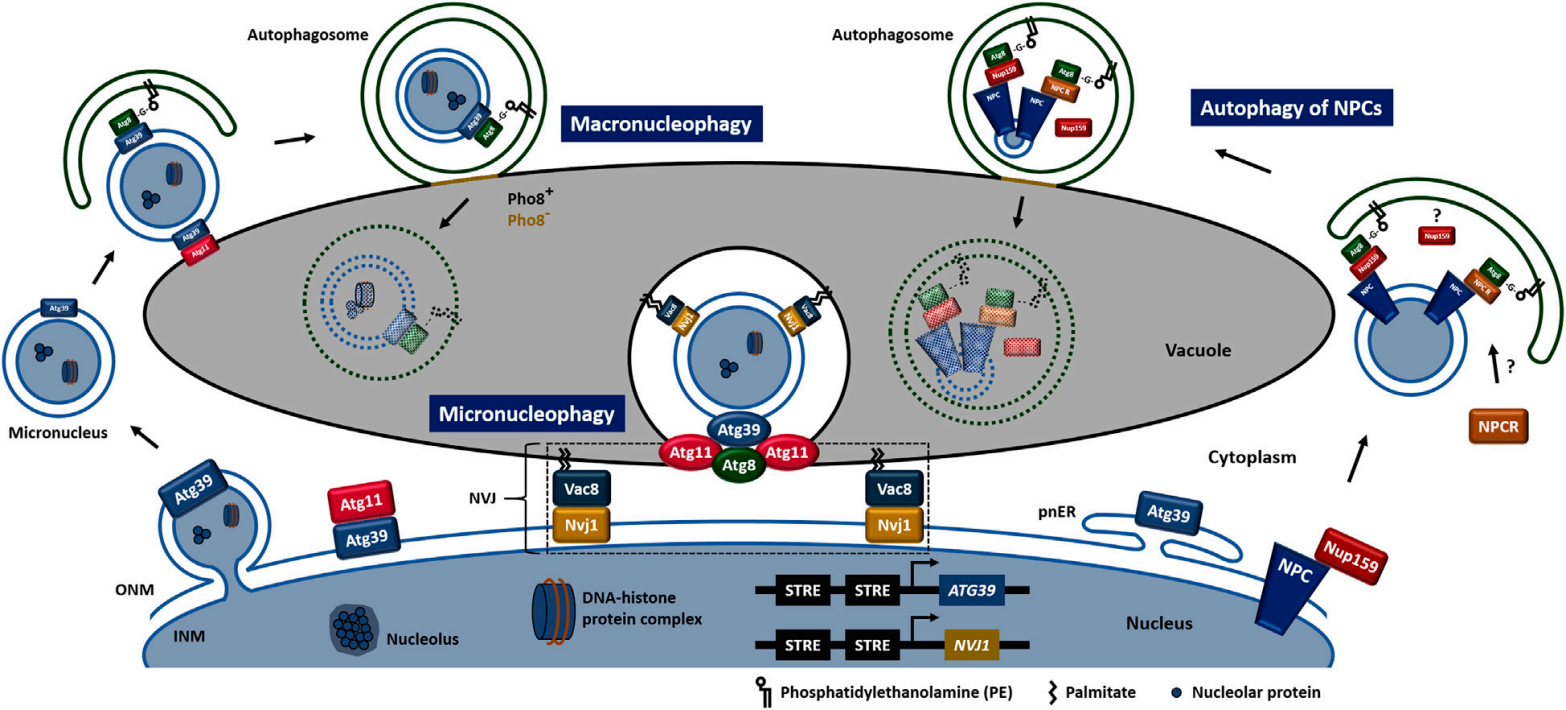
Nucleophagy in *S. cerevisiae*. Schematic models of macronucleophagy, micronucleophagy and autophagy of NPCs. Rectangular shapes of Atg8, Atg11, Atg39, NPCR, Nup159, Nvj1 and Vac8, outside of the nucleus represent single proteins. Oval shapes of Atg8, Atg11 and Atg39 represent microscopically-observed accumulation of the respective proteins. IMN, inner nuclear membrane; NPCR, nuclear pore complex unknown receptor; NPC, nuclear pore complex; NVJ, nucleus-vacuole junction; ONM, outer nuclear membrane; pnER, perinuclear endoplasmic reticulum.

In the budding yeast, *Saccharomyces cerevisiae*, both macronucleophagy and micronucleophagy have been reported (Figure 1). Carbon and nitrogen deprivation or pharmacological inactivation of the target of rapamycin complex 1 (TORC1) induce nucleophagy (Roberts et al., 2003; Mochida et al., 2015). During macronucleophagy the nuclear cargo receptor, autophagy related protein (Atg) 39, which localizes to both inner and outer nuclear membrane (INM and ONM, respectively), the perinuclear endoplasmic reticulum (ER), and also decorates macronucleophagy cargo, interacts with Atg8, a well-established marker of the autophagic membrane. The above receptor recognition results in micronuclei transfer/engagement to vacuole at an Atg11-positive site (Mochida et al., 2015; Otto and Thumm, 2021). Within its cytosolic N-terminal region, Atg39 contains both an Atg8-interacting motif (AIM) and an Atg11-binding region (11BR) enabling Atg39 to interact directly with Atg8 and Atg11 respectively. Fusion of autophagosome with vacuole is signified by a reduction in the integral vacuolar membrane protein, repressible alkaline phosphatase 8 (Pho8) indicating the initial change in the vacuolar membrane composition at the fusion site. Macronucleophagy substrates include the INM and ONM excluding the nuclear pore complexes, granular nucleolus, nucleolar proteins, spindle pole bodies, ribonucleic acid (RNA), pre-ribosomes and parts of the nucleoplasm excluding ribosomal DNA (Mostofa et al., 2018; Mostofa et al., 2019).

Micronucleophagy, also known as piecemeal microautophagy of the nucleus (PMN), is restricted to the nucleus-vacuole junction (NVJ), which is an inter-organelle contact site formed through the direct interaction between the C-terminus of the integral membrane protein, nucleus-vacuole junction protein 1 (Nvj1) and the vacuolar membrane protein, vacuolar protein 8 (Vac8) (Pan et al., 2000). An electrochemical gradient across the vacuolar membrane and lipid-modifying enzymes promote invagination of NVJs (Dawaliby and Mayer, 2010). Release of micronucleophagic vesicles into the vacuole lumen requires the vacuolar-adenosine triphosphatase (V-ATPase) activity. Micronucleophagy is characterized by Atg11- and Atg8-positive structures localized between the tips of the vacuolar invagination. Apart from the essential cargo receptor Atg39, other Atg proteins required for efficient micronucleophagy include the two ubiquitin-like conjugation systems, Atg9-cycling system, phosphoinositide 3-kinase (PI3K) complexes, cytoplasm-to-vacuole targeting (Cvt)-specific proteins and homotypic vacuole fusion proteins (Krick et al., 2008). Additional and exclusive micronucleophagy cargos include the NVJs and the vacuolar membrane.

Interestingly, both *NVJ1* and *ATG39* upstream promoter regions contain two putative stress-response element (STRE) repeats, subjecting the two proteins under common expression regulation (Moskvina et al., 1998; Gasch et al., 2000). In addition, Nvj1 and Atg39 localization depends on the nuclear envelope-localized phosphatase complex, nuclear envelope morphology protein 1/sporulation-specific protein 7 (Nem1/Spo7) and the phosphatidic acid phosphatase 1 (Pah1) upon TORC1 inactivation (Siniossoglou et al., 1998; Dubots et al., 2014; Rahman et al., 2018). The yeast Nem1/Spo7-Pah1 axis is conserved to mammalian cells, as the orthologous C-terminal domain (CTD) nuclear envelope phosphatase 1 (CTDNEP1)/nuclear envelope phosphatase regulatory subunit 1 (NEP1R1)-lipin complex which localises to the nuclear envelope as well (Kim et al., 2007). Hemizygous mutations of *CTDNEP1* were found in human medulloblastoma, a common type of primary brain cancer in children (Pugh et al., 2012).

### Autophagy of Nuclear Pore Complexes

Nuclear pore complexes (NPCs) are large protein channels penetrating the nuclear envelope, responsible for nucleocytoplasmic transport of various cellular components (Hampoelz et al., 2019). Expectedly, accumulating evidence show that disruption of NPC integrity is linked to ageing, cancer and neurodegenerative diseases (D'Angelo et al., 2009; Simon and Rout, 2014; Sakuma and D'Angelo, 2017). In yeast and *Caenorhabditis elegans*, age-dependent deterioration of nuclear pore assembly decreases transport dynamics and leads to cytoplasmic protein leak into the nucleus, respectively (D'Angelo et al., 2009; Rempel et al., 2019). In mouse and human neuronal cells, cytoplasmic mislocalization and aggregation of transactive response (TAR) DNA-binding protein-43 (TDP-43) disrupts nuclear pore complexes and furthermore nucleocytoplasmic transport in amyotrophic lateral sclerosis and frontotemporal dementia (ALS and FTD) disease spectrum (Chou et al., 2018). In human metastatic warm-autopsy prostate tumour tissues the nuclear envelope pore membrane protein 121 (POM121) was found upregulated (Rodriguez-Bravo et al., 2018). POM121 enhances importin-dependent nuclear transport of oncogenic and prostate cancer-specific transcription factors, promoting prostate cancer aggressiveness. Targeting the POM121-importin β axis was proposed as a therapeutic strategy for lethal prostate cancer.

Recently, scientists gained insights into the possible role of autophagy in NPC turnover (Figure 1). Upon nitrogen starvation turnover of NPCs involves vacuolar proteases and is mediated *via* the direct recognition of the cytoplasmically exposed nucleoporin 159 (Nup159) by Atg8 (Lee et al., 2020). Another study suggests that upon inactivation of TORC1 the above interaction is responsible for the degradation of the exact nucleoporin not assembled into the NPC, distinguishing “NPC-phagy” from “nucleoporinophagy” (Tomioka et al., 2020). Authors propose the existence of an unknown autophagy receptor for NPC-phagy.

### Complete and Unconventional Modes of Nucleophagy

Programmed nuclear death, programmed nuclear destruction and an uncharacterized mode of nucleophagy represent modes of nucleophagic degradation of the entire nucleus (Akematsu et al., 2010; Shoji et al., 2010; Eastwood et al., 2012; Liu and Yao, 2012; Corral-Ramos et al., 2015; Kikuma et al., 2017). However, the majority of those studies have been performed in multinucleate cells of filamentous fungi where one particular nucleus is selected among multiple nuclei and eliminated by nucleophagy without causing cell lethality. In yeast, after prolonged nitrogen starvation, late nucleophagy (LN) enables late delivery of nucleoplasm components to the vacuole, a distinct process from PMN that commences soon after nitrogen deprivation and requires nucleus-vacuole intermediates (Mijaljica et al., 2012).

### Nucleophagy in Mammals

In mammals, nucleophagy has mainly been monitored under pathological conditions, such as neurodegeneration and cancer (Table 1). Nuclear lamina, which is absent in yeast, is associated with mammalian INM, provides mechanical stability and supports a variety of nuclear activities such as chromatin organization, DNA replication, RNA transcription, cell cycle regulation, nuclear migration and apoptosis (Karoutas and Akhtar, 2021). Lamin A/C and lamin B, two of the major constituents of nuclear lamina have been identified as mammalian nucleophagic substrates (Dou et al., 2015).

**TABLE 1:** Nucleophagy in mammals.

Nucleophagic signaling	Cargo composition	Physiological/pathological setting	Reference
Nuclear envelopathies	DNA, H1, γ-H2AX	Nuclear damage response	Park et al. (2009)
Cell cycle perturbation	DNA, H2B, γ-H2AX, lamin B1	Genome stability	Rello-Varona et al. (2012)
Senescence	DNA	Stability of senescence/Tumour suppression	Ivanov et al. (2013)
Arginine starvation	DNA, NUP98	Prostate cancer cell death	Changou et al. (2014)
DNase deficiency/DNA damage	DNA	Cancer/Inflammation	Lan et al. (2014)
Oncogenic insult	DNA, lamin B1, H3K27me3	Cell/tissue integrity/Tumorigenesis restriction	Dou et al. (2015)
Keratinocyte differentiation	DNA, HP1a	Epidermal barrier function	Akinduro et al. (2016)
DRPLA	Lamin B1	Neuronal cell degeneration/death	Baron et al. (2017)
HGPS/progerin	DNA, progerin	Nuclear integrity	Lu and Djabali, (2018)
DNA damage	DNA, lamin A/C	Tumorigenesis restriction	Li et al. (2019)

Nucleophagy-inducing signaling and respective cargo recognition by the autophagic machinery under the context of mammalian physiology and pathology. γ-H2AX, serine-139 phosphorylated H2A histone family, member X; DNA, deoxyribonucleic acid; DNase, deoxyribonuclease; DRPLA, dentatorubral-pallidoluysian atrophy; H1, histone H1; H2B, histone H2B; H3K27me3, tri-methylated lysine-27 histone H3; HGPS, Hutchinson-Gilford progeria syndrome; HP1α, heterochromatin protein 1α; NUP98, nucleoporin 98.

Microtubule associated protein 1 light chain 3 (MAP1LC3, hereafter referred to as LC3), a mammalian homologue of Atg8, resides both in the cytoplasm and the nucleus (Drake et al., 2010). Upon starvation, LC3 undergoes nucleocytoplasmic transport and activation, through deacetylation by the nuclear-localized deacetylase sirtuin 1 (Sirt1) (Huang et al., 2015). However, on the one hand, such a transport probably represents a regulatory mechanism for LC3 subcellular distribution and availability upon nutrient starvation, rather than a nucleophagy-related process. On the other hand, bimolecular fluorescence complementation (BiFC) suggests that nuclear localized LC3 directly interacts with lamin B1 and indirectly to lamin-associated chromatin domains (LADs) at the site of nuclear lamina (Dou et al., 2015). This interaction is dependent on LC3 lipidation. Nucleus-to-cytoplasm transport and delivery of lamin B1, but not lamin A/C or B2, to lysosomes for degradation is mediated upon induced oncogenic rat sarcoma (*RAS*) gene in primary, but not immortalized, human cells. Either autophagy inhibition or LC3-lamin B1 interaction inhibition impair lamin B1 degradation and interestingly, attenuate oncogene-induced senescence in human cells (Dou et al., 2015). These findings suggest that autophagic degradation of a nuclear lamina component could protect cells from tumorigenesis.

DNA damage induces nuclear accumulation of the small ubiquitin-related modifier (SUMO) E2 ligase, ubiquitin conjugating enzyme E2 I/ubiquitin conjugating enzyme 9 (UBE2I/UBC9) in human MDA-MB-231 and MCF-7 breast cancer cell lines (Li et al., 2019). UBE2I/UBC9 SUMOylates lamin A/C and enables the interaction with LC3, which was required for nucleophagic degradation of lamin A/C and leaked nuclear DNA. Taking together, current findings suggest that lamins represent a prominent category of nuclear components able to integrate signaling from diverse nuclear insults and propagate nucleophagic signaling upon direct interaction with LC3.

Laminopathies, a group of diverse genetic disorders caused by mutations in proteins of the nuclear intermediate filament network have been linked to muscular dystrophy, neurodegeneration and premature ageing as well. Emerinopathies is a distinct group of disorders caused by mutations in the *EMD* gene encoding emerin, a nuclear envelope-anchored protein implicated in the regulation of transcription factor activity, cell signaling, nuclear architecture, chromatin condensation, organization, and epigenetic modification, nucleo-cytoskeletal mechanotransduction and cellular polarity organization (Koch and Holaska, 2014). Emerinopathies result in muscular dystrophies as well as cardiomyopathy and atrial fibrillation. Consequently, mutations or loss of lamins or emerin perturb nuclear architecture and cause nuclear membrane fragility especially in cells which are constantly subjected to mechanical stress such as skeletal and muscle cells (Sakaki et al., 2001; Mislow et al., 2002; Bengtsson and Wilson, 2004; Broers et al., 2004; Lammerding et al., 2004; Zhang et al., 2005). Lamins are a prominent target of nucleophagy during oncogenesis and neurodegeneration, resulting in cellular senescence and neuronal death, respectively. Perinuclear vacuolar structures were observed in skeletal and cardiac muscles of human and mouse nuclear envelopathies origin (a collective term for pathologies caused by mutations in the genes encoding lamins and emerin) (Park et al., 2009). These vacuolar structures represent autophagosomes/autolysosomes. Lamin A, lamin B and emerin are accumulated in the interface of those autophagic structures and the nuclear membrane. Moreover DNA, histone H1 and serine-139 phosphorylated H2A histone family, member X (γH2AX) are present into the perinuclear autophagosomes. γH2AX represents a *bona fide* marker of DNA double-strand breaks (Rogakou et al., 1998). Therefore, nucleophagy contributes to the rapid repair of multiple nuclear components. Similar structures were observed in wild type cells although to a much lower frequency, showing that nucleophagy can be utilized as a physiological response to conditions that cause spontaneous nuclear damage (Park et al., 2009).

A variety of cell cycle perturbations manifest increased numbers of cytosolic micronuclei and autophagosomes (Rello-Varona et al., 2012). A subpopulation of micronuclei, positive for chromatin and the histone H2B colocalize with green fluorescent protein (GFP) tagged LC3 in an ATG5- and ATG7-dependent manner, indicating that micronuclei can be subjected to autophagic degradation and ultimately contribute to genome stability. The autophagic sequestration of micronuclei was supported by electron microscopy analyses. Moreover, autophagic micronuclei found positive for the autophagic receptor p62/sequestosome 1 (SQSTM1), lamin B1 and the DNA damage marker γH2AX (Rello-Varona et al., 2012).

### Neurodegeneration

Autophagy, serving as a housekeeping quality control mechanism, is distinctly essential for long-living cells and frequently fails during neurodegeneration. In a mouse model of the polyglutamine disease, dentatorubral-pallidoluysian atrophy (DRPLA) as well as human fibroblasts of DRPLA patients, pathological manifestations include inhibition of LC3 lipidation, p62 accumulation and reduced transcription factor EB (TFEB) expression, rendering autophagy a stalled mechanism (Baron et al., 2017). Alternative clearance pathways including Golgi membrane-associated and nucleophagy-based lamin B1 degradation are activated in fibroblasts derived from DRPLA and Vici syndrome patients (Baron et al., 2017). The above results in dramatic nuclear breakdown and promotes terminal cell atrophy and death.

### Cancer

There are contradicting pieces of evidence regarding autophagy modulation and cancer treatment (Levy et al., 2017). Chemotherapy and radiotherapy as well as other currently available cancer treatments cause DNA damage and intervene autophagy indirectly. Autophagy induction has been shown to be beneficial when treating cancer at the beginning of the tumour establishment. Nucleophagic elimination of problematic genetic material, can maintain nuclear integrity and genomic stability during tumorigenesis. However, autophagy may promote cancer cell survival and metastasis in late stage tumour microenvironment where nutrient availability is limited.

In argininosuccinate synthetase 1 (ASS1)-deficient prostate cancer cells, arginine depletion induces giant autophagosome formation, nuclear membrane rupture, and interestingly cytoplasmic histone-associated DNA encaptured by autophagosomes (Changou et al., 2014). Thus, the fundamental process of metabolic stress-based cancer therapy could involve mechanisms related to DNA leakage, and chromatin autophagy. In both phagocytotic and non-phagocytotic cells which are either deficient for the lysosomal deoxyribonuclease 2a (Dnase2a) or deficient for autophagy, damaged DNA is exported from nucleus and accumulates in the cytosol (Lan et al., 2014). The above indicates that damaged chromosomal DNA could be physiologically cleared by autophagy. Failure of extranuclear DNA clearance induces inflammation *via* the stimulator of interferon response cyclic guanosine monophosphate-adenosine monophosphate (cGAMP) interactor (Sting)-dependent cytosolic DNA-sensing pathway.

### Differentiation

Upon terminal differentiation, keratinocytes migrate to the granular layer, lose organelles progressively and convert into anucleate corneocytes. Epidermal terminal differentiation is accompanied by activation of LC3, unc-51 like autophagy activating kinase 1 (ULK1), tryptophan-aspartate (WD) repeat domain, phosphoinositide interacting 1 (WIPI1), beclin 1 (BECN1) and ATG5-ATG12 expression in mouse fetal skin and detection of epidermal autophagic vesicles in new-born mouse epidermis (Akinduro et al., 2016). Interestingly, in monolayer keratinocyte cultures, terminal differentiation is accompanied by targeted autophagic degradation of nuclear material positive for the histone interacting protein, heterochromatin protein 1α (HP1α).

### Ageing

Nuclear size, architecture and genome copy number of the cells correlate with age and longevity. In certain premature-ageing syndromes, the nuclei adopt abnormal phenotypes and genome copy numbers (Tiku et al., 2017). Ribosomal RNA production increases with age as well. Patients with Hutchinson-Gilford progeria syndrome (HGPS), a rare genetic disorder characterized by dramatic and rapid development of ageing features in childhood, show expanded nucleoli, elevated global protein synthesis levels and enhanced ribosome biogenesis (Buchwalter and Hetzer, 2017). The above is caused by a spontaneous point mutation in the *LMNA* gene (encoding lamin A) leading to an aberrant splicing event (De Sandre-Giovannoli et al., 2003; Eriksson et al., 2003). Such a mutation generate a 50-amino acid in-frame deletion of prelamin A at its C-terminus, resulting in a permanently farnesylated and carboxymethylated protein termed progerin. Nuclear-localized reporter peptides bearing the toxic prelamin A and progerin C-terminal domains induce nucleophagic degradation accompanied by chromatin degradation (Lu and Djabali, 2018). In parallel, the Sad1 and UNC84 domain containing 1 (SUN1) protein, a constituent of the linker of nucleoskeleton and cytoskeleton (LINC) complex, delocalizes from the elongated nuclear envelope-autophagosome complex formed. Therefore SUN1 is excluded from nucleophagic degradation. Interestingly, the mutant messenger RNA (and therefore the mutant progerin protein expression) has been shown to be efficiently targeted and eliminated by sterically blocking the activated cryptic splice site using a morpholino oligonucleotide (Scaffidi and Misteli, 2005). On the other hand, small nucleolus size and decreased expression of ribosomal RNA, fibrillarin and ribosomal proteins are cellular hallmarks of longevity and metabolic health conserved across taxa (Yi et al., 2015; Buchwalter and Hetzer, 2017; Tiku et al., 2017; Tiku and Antebi, 2018). Age- or disease-related phenotypic changes of the nucleus are delayed or attenuated in long-lived *C. elegans* strains (Golden et al., 2007).

Senescent cells, damaged cells that permanently exit the cell cycle, shed chromatin fragments into the cytoplasm. Such cytoplasmic chromatin fragments (CCFs) are strongly positive for histones H2 and H3 and negative for lamin A/C (Ivanov et al., 2013). CCFs have been associated with lamin B1 down-regulation and furthermore loss of nuclear envelope integrity. In addition, CCFs are targeted by the autophagic machinery to lysosomes. Increasing interest in CCF formation, function and elimination advances our understanding of nuclear autophagic events but also genome integrity and senescence-associated inflammation (Miller et al., 2021). Therefore, the study of cytoplasmic DNA, histone and nuclear lamina species can contribute to therapies aiming to delay senescence and improve human health.

## Conclusion and Perspectives

Although research interest in nucleophagy is emerging, several aspects of mammalian nucleophagy remain still elusive. These include firstly, the morphological and spatiotemporal membrane dynamics and secondly the molecular targeting and mechanisms of selectivity of nucleophagy. Unlike the most of the selective types of autophagy, nucleophagy must target and process only a select portion of the organelle. Similar to ER-phagy, macronucleophagy involves bulging of the nuclear envelope and budding of the micronucleus. Among the ER-resident proteins that have been shown to function as selective receptors for ER-phagy, reticulophagy regulator 1 (RETREG1)/family with sequence similarity 134, member B (FAM134B) which contains a reticulon homology domain (RHD), conveys membrane-curvature induction and curvature-mediated protein sorting required for ER-phagy (Bhaskara et al., 2019). Atg39, the nuclear membrane resident protein that functions as selective receptor for nucleophagy, does not contain an RHD. It is possible that non-characterized curvature inducing factors that localize to the ONM trigger the initial bulging of the nuclear envelope and facilitate micronuclei formation.

Extensive characterization of the nucleophagic content and membrane composition will advance our understanding and reveal the causal basis of nucleophagic events. It is not clear whether nucleoplasm and nucleoplasmic or nucleolar proteins are actual and selective targets of nucleophagy. It is possible that their degradation is circumstantial or serves as a concomitant low-level basal turnover mechanism. Interesting topics of investigation include the nucleophagy regulatory mechanisms, the identification of the participating receptors and therefore the underlying coordination and substrate specificity in health, disease and ageing. It is conceivable that sequestering genetic or nuclear material and routing them for degradation should obey delicate and strict regulation. If so, identification of possible unique mechanisms, markers and receptors could lead drug discovery to highly selective pharmacological interventions towards the preservation of insulted nuclear integrity.
